# A systematic review of non-coding RNA genes with differential expression profiles associated with autism spectrum disorders

**DOI:** 10.1371/journal.pone.0287131

**Published:** 2023-06-15

**Authors:** Jon Stott, Thomas Wright, Jannah Holmes, Julie Wilson, Sam Griffiths-Jones, Deborah Foster, Barry Wright

**Affiliations:** 1 Child Oriented Mental Health Intervention Collaborative (COMIC), University of York in Collaboration with Leeds and York Partnership NHS Foundation Trust, York, United Kingdom; 2 Tees, Esk & Wear Valleys NHS Foundation Trust, Foss Park Hospital, York, United Kingdom; 3 Manchester Centre for Genomic Medicine, Clinical Genetics Service, Saint Mary’s Hospital, Manchester University NHS Foundation Trust, Manchester, United Kingdom; 4 Division of Evolution, Infection and Genomics, School of Biological Sciences, Faculty of Biology, Medicine and Health, University of Manchester, Manchester, United Kingdom; 5 Hull York Medical School, University of York, Heslington, York, United Kingdom; 6 Department of Mathematics, University of York, Heslington, York, United Kingdom; Universita Politecnica delle Marche, ITALY

## Abstract

**Aims:**

To identify differential expression of shorter non-coding RNA (ncRNA) genes associated with autism spectrum disorders (ASD).

**Background:**

ncRNA are functional molecules that derive from non-translated DNA sequence. The HUGO Gene Nomenclature Committee (HGNC) have approved ncRNA gene classes with alignment to the reference human genome. One subset is microRNA (miRNA), which are highly conserved, short RNA molecules that regulate gene expression by direct post-transcriptional repression of messenger RNA. Several miRNA genes are implicated in the development and regulation of the nervous system. Expression of miRNA genes in ASD cohorts have been examined by multiple research groups. Other shorter classes of ncRNA have been examined less. A comprehensive systematic review examining expression of shorter ncRNA gene classes in ASD is timely to inform the direction of research.

**Methods:**

We extracted data from studies examining ncRNA gene expression in ASD compared with non-ASD controls. We included studies on miRNA, piwi-interacting RNA (piRNA), small NF90 (ILF3) associated RNA (snaR), small nuclear RNA (snRNA), small nucleolar RNA (snoRNA), transfer RNA (tRNA), vault RNA (vtRNA) and Y RNA. The following electronic databases were searched: Cochrane Library, EMBASE, PubMed, Web of Science, PsycINFO, ERIC, AMED and CINAHL for papers published from January 2000 to May 2022. Studies were screened by two independent investigators with a third resolving discrepancies. Data was extracted from eligible papers.

**Results:**

Forty-eight eligible studies were included in our systematic review with the majority examining miRNA gene expression alone. Sixty-four miRNA genes had differential expression in ASD compared to controls as reported in two or more studies, but often in opposing directions. Four miRNA genes had differential expression in the same direction in the same tissue type in at least 3 separate studies. Increased expression was reported in *miR-106b-5p*, *miR-155-5p* and *miR-146a-5p* in blood, post-mortem brain, and across several tissue types, respectively. Decreased expression was reported in *miR-328-3p* in bloods samples. Seven studies examined differential expression from other classes of ncRNA, including piRNA, snRNA, snoRNA and Y RNA. No individual ncRNA genes were reported in more than one study. Six studies reported differentially expressed snoRNA genes in ASD. A meta-analysis was not possible because of inconsistent methodologies, disparate tissue types examined, and varying forms of data presented.

**Conclusion:**

There is limited but promising evidence associating the expression of certain miRNA genes and ASD, although the studies are of variable methodological quality and the results are largely inconsistent. There is emerging evidence associating differential expression of snoRNA genes in ASD. It is not currently possible to say whether the reports of differential expression in ncRNA may relate to ASD aetiology, a response to shared environmental factors linked to ASD such as sleep and nutrition, other molecular functions, human diversity, or chance findings. To improve our understanding of any potential association, we recommend improved and standardised methodologies and reporting of raw data. Further high-quality research is required to shine a light on possible associations, which may yet yield important information.

## Introduction

Autistic people are thought to account for at least 1% of the global population [1]. Individuals with a diagnosis of autism have differences in social communication and are more likely to have intense interests [2–4]. People with autism belong within a spectrum of neurodiversity that is important for society and evolution [5]. For the purpose of this systematic review we have followed the established international diagnostic criteria and the corresponding nomenclature [6]. From herein we will use the associated terminology, autism spectrum disorder (ASD), although we acknowledge that different perspectives exist regarding language and terminology preferences [7–9]. The genomic landscape of ASD is complex [10], however a strong genetic aetiology is recognised [11, 12] with twin studies estimating heritability between 70–90% [13, 14]. Access to broad genomic testing is reshaping our understanding of ASD, which appears to encompass a collection of broad, heterogenous [15] and variable conditions with overlapping neurobehavioral phenotypes [16]. These may be considered on one hand as complex or syndromic when ASD symptomatology features alongside intellectual disability, facial dysmorphism or congenital malformations [17]. On the other hand, non-syndromic ASD symptomatology may comprise a broader understanding of neurodiversity [5]. High impact genetic variants are reported to occur in around 15% of individuals with ASD, which are predominantly caused by nuclear sequence-level and structural variants, or less commonly mitochondrial variants [18]. It is important to recognise the variable contribution genetic variants have made towards ASD symptomatology, which frequently demonstrate incomplete penetrance and variable expressivity [19].

Proposed explanations for the high heritability, but low monogenic diagnostic findings in ASD include oligogenic and polygenic models of aetiology [20]. Other proposed genetic aetiologies include the imprinted brain theory where there is a paternal bias in the expression of imprinted genes [21] and epigenetic contribution [22]. Given that most nucleotides in the human genome are outside of open reading frames of protein coding genes [23], yet around 75% of the genome are transcribed [24], this draws our attention inexorably to non-coding RNA transcripts that comprise functional molecules that may play an important role in gene expression and gene-environment interactions in ASD. A good starting point is a synthesis of the ncRNA gene expression literature to delineate further promising avenues of enquiry for ASD research [25].

### Classification of non-coding RNA

ncRNA are described in detail elsewhere [26, 27]. They have historically been categorised by size, where long non-coding RNA (lncRNA) are 200 or more nucleotides and short ncRNA are less than 200 nucleotides in length [28]. The terms “short” or “small” however, are being used less to describe ncRNA, and do not feature in the current approved nomenclature [26]. Many ncRNA molecules regulate gene expression via RNA interference, epigenetic modification and inhibition of translation related mechanisms [29]. Secreted extracellular circulating ncRNA are, in many cases, highly stable and detectable in multiple biological fluids such as blood, saliva and urine [30, 31]. There is great interest in developing ncRNA expression assays translatable into a clinical setting that may be capable of supporting ASD diagnostics and providing phenotypic or prognostic information to enhance ASD care [32, 33]. The HUGO Gene Nomenclature Committee (HGNC) have worked with specialist advisors to define the accepted nomenclature for ncRNA [26]. HGNC define nine major classes of ncRNA annotated in the human genome. In this systematic review, we are interested in the shorter classes ncRNA and their relative gene expression in ASD. We are not considering genomic variation within ncRNA genes [34, 35], or the expression of larger ncRNA such as ribosomal RNA [36, 37] or long non-coding RNA (lncRNA) [38, 39]. The shorter ncRNA HGNC approved gene classes included in this systematic review are: microRNA (miRNA), piwi-interacting-RNA (piRNA), small NF90 (ILF3) associated RNA (snaR), small nuclear RNA (snRNA), small nucleolar RNA (snoRNA), transfer RNA (tRNA), vault RNA (vtRNA), and Y RNA. They have been summarised in Table 1. Whilst we acknowledge that HGNC approval is only in place for piRNA gene clusters, given the likely expansion to include individual piRNA genes in the future and given that annotation exists elsewhere [40], they have also been included. For simplicity, we will collectively refer to the shorter ncRNA classes included in this systematic review as ncRNA from herein.

**Table 1:** Shorter HGNC approved ncRNA gene classes included in this systematic review.

Name (abbreviated name)	HGNC gene symbols^#^	Nucleotide length	Cellular functions	Database / key references
MicroRNA (miRNA)	1912	21–24	Regulation of post-transcriptional gene expression by complementary mRNA binding that mediates translational repression or mRNA degradation	miRBase v22.1 [49]; Bartel., 2018 [50]
PIWI-interacting RNA* (piRNA)	114 clusters	24–31	Predominantly germline expressed that silence transposable elements, regulate gene expression and counteract viruses by RNA cleavage, DNA methylation and heterochromatin assembly	piRBase v3.0 [40]; Ozata et al., 2019 [51]
Small NF90 (ILF3) associated RNA (snaR)	28	117	Abundantly expressed in the testes, placenta, and discrete regions of the brain with tissue specific regulation of cellular growth and translation	Parrott et al., 2011 [52]
Small nuclear RNA (snRNA)	50	150	Components of the major and minor spliceosome complexes to splice introns from pre-messenger RNA	Karijolich & Yu., 2010 [53]; Ma et al., 2022 [54]
Small nucleolar RNA (snoRNA)—three types: i. C/D box (SNORD); ii. H/ACA box (SNORA); iii. Small Cajal body-specific RNA (scaRNA)	568	30–300	Guide RNAs for post transcriptional modification and maturation of ribosomal RNA and small nuclear RNA by methylation and pseudouridylation.	snoRNABase** [55]; Bratkovič et al., 2019 [56]
Transfer RNA (tRNA)	591	73–93	Protein translation of mRNA on the ribosome	GtRNAdb 2.0 [57]
Vault RNA (vtRNA)	4	88–100	Form large and highly conserved ribonucleoprotein complexes implicated in autophagy, apoptosis, and cell proliferation	Büscher et al., 2020 [58]
Y RNA	4	100	Bound by Ro60 and La proteins, with roles in DNA replication, RNA stability and cellular stress responses	Kowalski & Krude., 2015 [59]; Valkov & Das., 2020 [60]

**Table Footnote:** #available from https://www.genenames.org/download/statistics-and-files/ [61]

* individual piRNA genes listed in piRBase v3.0 were included in this systematic review, as only piRNA gene clusters have current approval by HGNC; **snoRNABase is no longer updated, but it remains a useful resource. Abbreviation mRNA = messenger RNA.

### Rationale for systematic review

A systematic review is warranted for a few key reasons. Firstly, much of the early research examining ncRNA expression profiles in association with ASD examines miRNA alone [41]. We may be missing other important classes of ncRNA. To our knowledge there has been no systematic review exploring gene expression of other ncRNA. Secondly, a large proportion of early research in this field is from post-mortem samples from brain tissue [42, 43]. These are important for discovery but may lack clinical translatability. To realise the potential of ASD ncRNA gene expression assays for biomarker use, we require an appreciation of the combined expression data from living patients with ASD from clinically available samples. To date there have been some narrative, discursive, selective or scoping reviews [25, 42, 44–48] and just one recent systematic review that only examines miRNA expression associated with ASD that is missing some studies [41]. Finally, in view of the recent international nomenclature describing ncRNA with HGNC approved human genome annotation [26], we are keen to collate and present up to date and standardised ncRNA gene expression data associated with ASD. We acknowledge that there may be a paucity of evidence for classes of ncRNA other than miRNA, but demonstrating and delineating this clearly by systematic review is important to help shape future research directions.

## Methods

PROSPERO registration number: CRD42020208233.

### Study eligibility criteria

The inclusion criteria were as follows:

Population: Human subjects with a diagnosis of ASD compared with controls without ASD.

Exposure: ncRNA gene expression profiles from biosamples measuring HGNC approved ncRNA genes or piRNA genes listed in piRBase v3.0.

Outcome(s): Expression profile of any of the following ncRNA genes: miRNA, piRNA, snaR, snRNA, snoRNA, tRNA, vtRNA, and Y RNA; using validated scientific methodologies.

Studies: Peer reviewed publications, conference abstracts or dissertations.

The exclusion criteria were as follows: studies not published in English, duplicated data, non-human studies, review articles, hypothesis papers, narrative reviews, fact sheets and letters to the editor that did not present unique or new data, unpublished materials and studies published before 2000.

### Search strategy

The following electronic databases were searched: Cochrane, EMBASE, Science Direct, Medline, PubMed, Scopus, Web of Science, PsychInfo, ERIC, AMED, and CINAHL. We searched databases from January 2000 to May 2022. Medical Subjective Heading (MeSH) search terms were used for autism spectrum conditions including ‘autism’, ‘autistic’, autism spectrum disorder, ‘ASD’, autism spectrum condition (ASC), ‘Asperger’, ‘pervasive developmental disorder’ and ‘PDD’ in all combinations with the terms ‘short non coding RNA’, ‘non-coding RNA’, ‘RNA’, ‘miRNA’, ‘miRNA’, ‘piwi interacting RNA’, ‘piRNA’, ‘ribosomal RNA’, ‘rRNA’, ‘small NF90 associated RNA’, ‘small NF90 (ILF3) associated RNA’, ‘snaRs’, ‘small nuclear RNA’, ‘snRNA’, ‘small nucleolar RNA’, ‘snoRNA’, ‘transfer RNA’, ‘tRNA’, ‘vault RNA’, and ‘Y RNA’. The references cited in identified publications were also searched to locate additional studies. Data related to ncRNA expression profiles was extracted where available, including information related to normalisation strategies, ncRNA gene expression fold change, P values and confidence intervals. Given the varied nomenclature used for ncRNA, gene names will be recorded together with HGNC codes, accession IDs from miRBase database v22.1 (mirbase.org) or piRBase database v3.0 (bigdata.ibp.ac.cn/piRBase).

### Procedure

Two reviewers independently screened the titles and abstracts to identify all eligible studies identified by the searches. Any discrepancies were adjudicated by a third reviewer. The reference lists of selected articles were used to identify additional papers for screening. The included studies were assessed using the Preferred Reporting Items for Systematic Review and Meta-Analysis (PRISMA) guidelines [62]. Data extraction took place and was recorded in a dedicated data extraction form generated using Microsoft Excel for further evaluation of study quality and data synthesis including functional enrichment analysis of the significant differentially expression miRNA genes. Raw data was retrieved from published papers, supplementary materials or by contacting the corresponding authors.

### Data synthesis and quality assessment

We planned to perform meta-analysis of ncRNA gene expression using the statistical techniques employed by Zhu and Leung [63], including a random effects model [64] to examine differentially expressed ncRNA genes in ASD compared with controls. We expected between study heterogeneity and subgroup analysis were to be used to explore possible sources, including source of patients, source of control (such as healthy control or disease control), participant ethnicity, ncRNA profile (single ncRNA and multiple ncRNA) and sample specimen (blood, saliva, urine, cultured lymphoblastoid cells, fibroblast cells, neural tissues, and others); living or post-mortem. We planned to analyse the statistical heterogeneity of the meta-analysis by x-squared (x^2^)-based Q statistic test when I^2^ (I-squared or I2) exceeded 50% or P < 0.1. Receiver-operating characteristics (ROC) curves were planned to be generated with sensitivity, specificity and positive predictive values based on known assessments of participants with ASD or without ASD. The area under the curve (AUC) was planned to be calculated both overall and for any subgroup analysis. Statistical tests were intended to be two-sided, with P < 0.05 considered statistically significant. Functional enrichment analysis of statistically significant differentially expressed miRNA genes as determined by meta-analysis would be performed using DIANA-miRPath v3.0 [65] and executed using the online DIANA-microT-CDS web-server algorithm to examine Gene Ontology (GO) with ‘categories union’. P-value and microT thresholds would be set at < 0.05 and 0.8, respectively with False Discovery Rate (FDR) correction applied. Targeted pathways and significance clusters will be generated and a related heatmap constructed.

We planned an assessment of publication bias [66] using Egger’s graphical test to construct a funnel plot of all studies included in the meta-analysis and explore the symmetry of the study distribution on the plot [64]. Begg and Mazumdar’s Rank Correlation test would be used to correlate the ranks of effect sizes and the ranks of their variances [67] and Orwin’s Fail-Safe N test would determine the presence of missing studies that may skew the regression line in the funnel plot, with Duval and Tweedie’s Trim and Fill method being used for imputation of the missing studies [68, 69]. The methodological quality of all included studies was assessed by two reviewers independently using a quality assessment template based on Quality Assessment of Diagnostic Accuracy Studies (QUADAS-2) [70].

## Results

### Studies identified for selection

**Fig 1:**
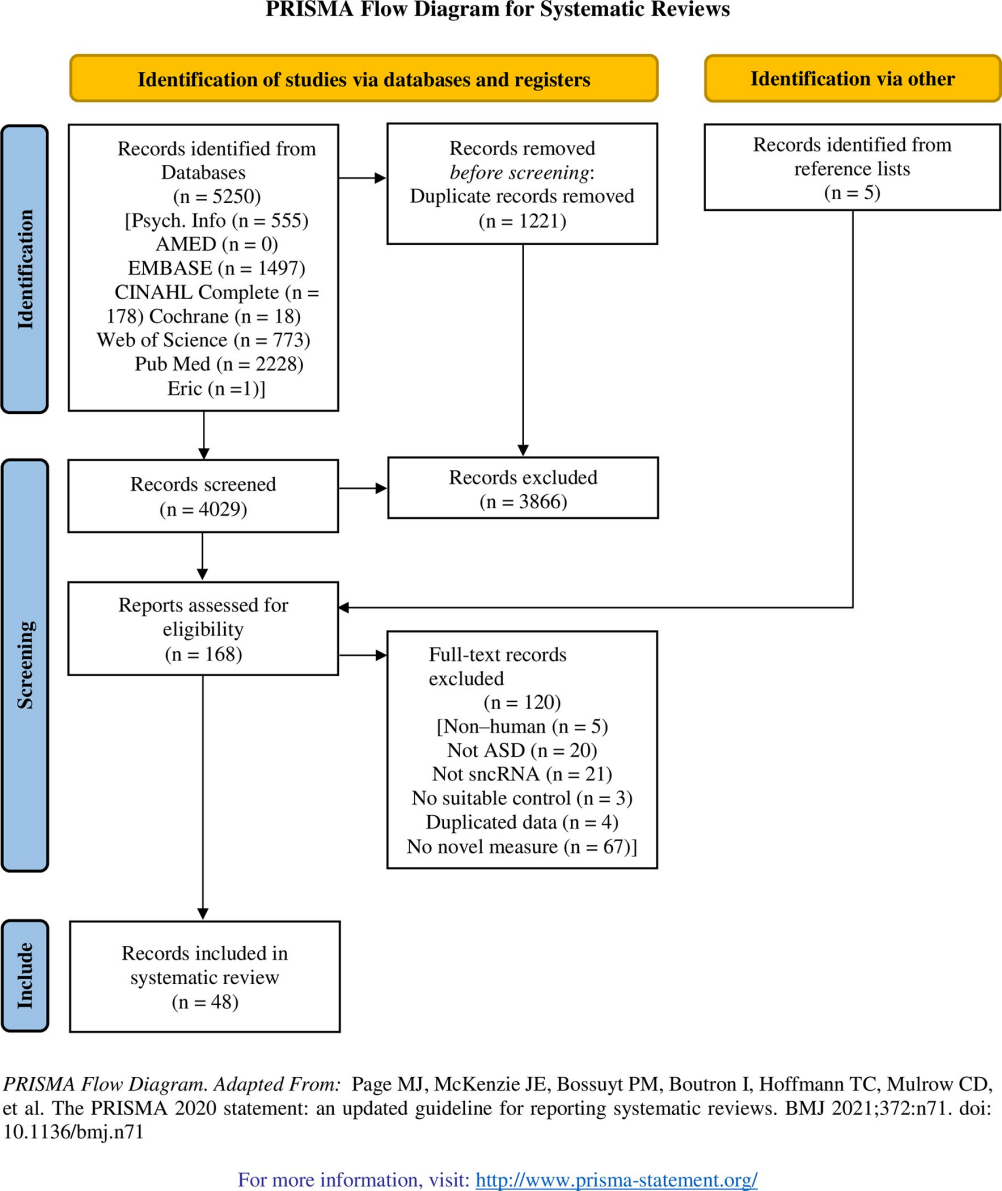
PRISMA flow chart illustrating the process of study selection.

The systematic review search strategy yielded 5250 publications, with 1221 being duplications. The titles and abstracts of 4029 papers were screened and 168 papers were assessed in full for eligibility. 48 studies were identified for inclusion in the systematic review for data extraction. This process is outlined along with reasons for exclusion in the PRISMA flow chart (Fig 1).

### Summary of eligible studies

This systematic review has brought together the findings of 48 studies involving over 1800 individuals with ASD compared with over 1400 controls. The year of publication ranged from 2008 to 2021. ASD ncRNA gene expression studies have been conducted in numerous countries across the world, including Brazil, Bulgaria, China, Egypt, Iran, Italy, Japan, United Kingdom, and United States of America (USA). The most prolific country for publication was the USA with 12 studies. Considering all included studies, the diagnosis of ASD of study participants in 16 studies reported the use of both a validated assessment tool and Diagnostic and Statistical Manual of Mental Disorders (DSM-5) criteria. There were 14 studies that only reported the use of a validated assessment tool, the most common being the Autism Diagnostic Interview-Revised (ADI-R) and the Autism Diagnostic Observation Schedule (ADOS). Eight studies solely used The World Health Organisation (WHO) or DSM diagnostic criteria without a validated assessment tool and 10 studies did not state the method of ASD diagnosis. The vast majority of studies (N = 46) examined miRNA gene expression; 41 studies did so exclusively and 7 studies examined other classes of ncRNA, of which 5 studies also measured miRNA gene expression (including a genome wide study ncRNA expression study encompassing miRNA genomic loci). Fourteen studies used pre-selected candidate-driven ncRNA expression approaches, for example where specific miRNAs had been investigated, in contrast to 34 studies that investigated unselected or larger populations of ncRNA genes including those examined using genome wide approaches. Many of these studies went on to examine (‘validate’) a selected population of miRNA genes identified by an initial unselected approach such as microarray or from RNA-seq. Thirty-three studies reported ncRNA expression findings using tissue samples and laboratory methodologies that could feasibly be implemented into clinical practice (i.e., those from living individuals, with routine sampling methodology of easily obtainable tissue such as blood or saliva and routine laboratory work). These studies had a male to female ratio of participants of 3.5 to 1. There were 15 studies that exclusively reported findings from studies with less or unfeasible clinical implementation possibilities (i.e., when samples derived from post-mortem brain tissue or studies from living individuals requiring specialist sampling procedures such as biopsies, or those with complex or time-consuming laboratory work such as cell culturing). These studies had a male to female ratio of participants of 4.8 to 1. There were two studies that examined ncRNA expression from both clinically feasible and unfeasible samples.

### Characteristics of eligible studies

Table 2 provides a summary of 33 studies describing methods feasible for clinical implementation. Of these, 29 reported ncRNA gene expression from peripheral blood and 4 reported from saliva samples. We found no studies exploring ncRNA gene expression from other bodily fluids such urine or sweat. Table 3 summarises the studies with less or unfeasible clinical implementation. From these 17 studies, 10 were from post-mortem brain tissue samples, five were from cultured lymphoblastoid cell lines, one was from reprogrammed induced pluripotent stem cell-derived neurons, and a further study reporting both olfactory mucosal stem cells and primary skin fibroblasts [71]. Two of these studies examined ncRNA gene expression from both clinically feasible and unfeasible samples [71, 72], and therefore feature in both Tables 2 and 3. Table 4 provides an overview of the individual ncRNA genes (all of which are miRNA genes) that have been reported to have increased or decreased expression in ASD cohorts in more than one study. The individual miRNA genes are listed with the direction of expression change presented by broad tissue sample types: blood, saliva, cultured lymphoblastoid cells (unless otherwise specified) and post-mortem brain samples. The seven studies examining differential expression of ncRNA genes other than miRNA have been presented in a separate table (Table 5).

**Table 2:** Studies examining ncRNA gene expression in ASD using tissue samples and laboratory methodologies that could be feasibly implemented into clinical practice (N = 33).

Study	Tissue sample	Diagnosis of ASD		Participants				Sample processing		Analysis	
		WHO / DSM criteria	Validated Ax tool	ASD		Control		RNA extraction	ncRNA assay	DEx	Normalisation strategy
				M:F ratio	Mean age	M:F ratio	Mean age				
Abdelrahman et al., 2021 [89]	Blood: whole	Yes	Yes	29:11	4 = median	22:8	3.6 = median	miRNeasy kit (Qiagen)	2 miRNA gene qPCR with TaqMan RT kits on QuantStudio 12K Flex (Applied Biosystems)	2^–ΔΔCt^ P < 0.05	*miR-16*
Atwan et al., 2020 [90]	Blood: PBMC	Not stated	Not stated	26:11	7	27:13	9	TRIzol method	2 miRNA gene qPCR with RealQ Plus 2x Mastermix Green (Ampliqon) on Rotorgene Q (Qiagen)	2^–ΔΔCt^ P < 0.05	*miR-16*
Cheng et al., 2020 [84]	Blood: NOS	Yes	Yes	14:9	8	Not stated N = 23	Not stated	TRIzol method	20 ncRNA gene assay using GenePix microarray and scanner (Axon Instrument)	Custom formula	Not stated
Cirnigliaro et al., 2017 [91]	Blood: serum	Yes	Yes	N = 4	-	N = 3	-	miRNeasy kit (Qiagen)	754 miRNA genes examined by TaqMan Low Density Array	2^–ΔΔCt^ FDR = 0.15 P < 0.05	Customised normalisation using top three stable miRNAs
				22:8	6.5	16:9	9.5		4 miRNA gene qPCR with TaqMan MicroRNA Assays (Applied Biosystems)		*miR-146a*
Cui et al., 2021 [92]	Blood: Serum	Yes	Not stated	142:17	3.13	137:22	3.3	TRIzol method	3 miRNA gene qPCR with SYBR Primix Ex Taq TM II (Takara) on ABI StepOne Plus (Applied Biosystems)	2^–ΔΔCt^ P < 0.05	Not stated
Eftekharian et al., 2019 [93]	Blood: whole	Yes	Yes	38:12	6	37:13	6.04	Hybrid-RTM blood RNA extraction kit (GeneAll)	4 miRNA gene qPCR with Applied Biosystems TaqMan Universal PCR Master Mix on Rotor Gene 6000 Corbett	2^–ΔΔCt^ P < 0.05	*RNU6-6P (RNU6B)*
Gao et al., 2021 [94]	Blood: whole	Not reported by Gao et al., 2021 [94].		GEO dataset: GSE18123				RiboPure blood kit (Ambion)	Affymetrix Gene 1.0 ST (GeneST) / Affymetrix U133 Plus 2.0 GeneChip arrays. Weighted gene co-expression network analysis	Bioconductor limma package P < 0.05	Quantile normalization using RMA
				66:0	7.9	33:0	9				
				GEO dataset: GSE6575				PAXgene Blood RNA System			
				30:0	2–5 = range	9:0	2–5 = range				
Hicks, Ignacio et al., 2016 [95]	Saliva	Yes	Yes	19:5	9.1	16.5	9.2	TRIzol method and miRNeasy kit (Qiagen)	246 miRNA genes examined by Illumina MiSeq with targeted depth of 3 million reads per sample	P < 0.05 FDR <0.15	Reads per million
Hicks, Carpenter et al., 2020 [96]	Saliva	Yes	Yes	161:26	4.5	76:50	3.9	TRIzol method and miRNeasy kit (Qiagen)	527 miRNA genes examined by Illumina TruSeq Small RNA Sample Prep by NextSeq500 instrument with targeted depth of 10 million reads per sample	FDR < 0.05 and/or PLS-DA ≥ 2.0	Read counts quantile-normalised, mean-centred, and divided by the standard deviation of each variable
						48:21	4.1				
Hirsch et al., 2018 [97]	Blood: NOS	Yes	Yes	N = 7	Not stated	N = 7	Not stated	TRIzol method	16 miRNA genes examined with SYBR Green qPCR on Applied Biosystems StepOne System	2^–ΔΔCt^	geNorm approach
Huang et al., 2015 [98]	Blood: NOS	Yes	Not stated	4:1	4.9	5	Not stated	TRIzol method	All miRNA genes from miRBase v20.0 by RiboArray miDETECT Human Array	2^–ΔΔCt^ Rank Product Method P < 0.01 (array), P < 0.05 (qPCR)	Quantile normalisation method
				13:2	4.3	Not stated: Sex and age matched			5 miRNA genes examined by qPCR on CFX96 BioRad		*miR-16-5p*
Jyonouchi et al., 2017 [99]	Blood: PBMC	Not stated	Yes	52:16	11.8 = median	16:11	10.1 = median	miRNeasy kit (Qiagen)	Ion Total RNA-Seq Kit V2, Ion Xpress RNA-Seq Barcode 1–16 kit and Ion One Touch 2 system using Ion 318 chips. Sequence reads processed in Torrent Server v4.4 (Life Technologies)	Analysis in Strand NGS 2.7. Fold change ≥ 2.0	Not stated
Jyonouchi et al., 2019 [100]	Blood: serum	Not stated	Yes	88:17	10.6 = median	27:8	13.3 = median	Not stated	Small RNA Library Prep Kit for Illumina (Norgen Biotek) on Illumina NextSeq 550 with 75 PCR cycles	Log 2 fold change	Read per million. Weighted trimmed mean of the log expression ratios (‘TMM’)
Kichukova, Popov et al., 2017 [76]	Blood: serum	Yes	Yes	20:6	6.86	20:6	Age matched	PAXgene blood miRNA kit (PreAnalytiX)	42 miRNA genes by qPCR with Maxima SYBR Green qPCR Master Mix (Тhermo Fisher) on ABI 7500 system (Applied Biosystems)	2^–ΔΔCt^	Spiked in *cel-miR-39*
Kichukova, Petrov et al., 2021 [101]	Blood: serum	Yes	Yes	30:8	Not stated	22:6	Not stated	PAXgene Blood miRNA kit (PreAnalytiX)	4 miRNA genes by qPCR with Maxima SYBR Green qPCR Master Mix (Тhermo Fisher) on ABI 7500 system (Applied Biosystems)	2^–ΔΔCt^	Spiked in *cel-miR-39*
Nakata et al., 2019 [102]	Blood: NOS	Not stated	Yes	18:12	28.4	18:12	28.4	Paxgene Blood RNA and miRNA System (Qiagen).	Agilent SurePrint G3 Human GE v2 8x60K Microarray (G4851B) (Agilent Technologies) followed by targeted qPCR of *miR-6126*	2^–ΔΔCt^ FDR < 0.05 Fold change > 1.25 P < 0.01	Spiked in *cel-miR-39*
Nguyen, Lepleux et al., 2016 [71]	Olfactory mucosal cells	Yes	Not stated	Not stated N = 8	Not stated	Not stated N = 6	Not stated	mirVana miRNA isolation kit (Life Technologies) / TRIzol method and miRNeasy kit (Qiagen)	667 miRNA genes from miRBase v10 using TaqMan Arrays A and B. Validation of target miRNAs using SYBR Green Power Mix (Life Technologies) by qPCR-HD-GPC platform (Paris)	2^–ΔΔCt^	*miR-221*
	Primary skin fibroblasts			4:1	5–41 = range	Not stated N = 4	Not stated				
	Blood: PBMC			8:1	5–18 = range	Not stated N = 20	Not stated				
Ozkul et al., 2020 [103]	Blood: serum	Yes	Not stated	31:14	2–13 = range	10:11	3–16 = range	High Pure miRNA Isolation Kit (Roche)	372 miRNA genes (384HC miScript miRNA PCR Array) using TaqMan Universal PCR Master Mix with qPCR on BioMark system (Fluidigm)	2^–ΔΔCt^	*SNORD61*, *SNORD68*, *SNORD72*, *SNORD95*, *SNORD96A*, *RNU6-2*, *miRTC*, and *PPC*
Pagan et al., 2017 [72]	Post-mortem pineal gland	Yes	Yes	8:1	39 = median	20:2	36.5 = median	mirVana PARIS kit (Ambion)	Single miRNA gene examined by qPCR (machine not specified) using miScript reverse transcription kit and SYBR Green PCR kit (Qiagen)	2^–ΔΔCt^ P < 0.0001	Spiked in *cel-miR39*, *cel-miR54* and *cel-miR238* (Qiagen)
	Blood: plasma			Not stated N = 54	Not stated	70	Not stated				
Popov, Minchev et al., 2018 [77]	Blood: serum	Yes	Yes	24:6	3–11 = range	30 sex matched	Age matched	PAXgene blood miRNA kit (PreAnalytiX)	Single miRNA gene examined by qPCR using Maxima SYBR Green qPCR Master Mix (Thermo Scientific) on ABI PRISM 7500 system (Applied Biosystems)	2^–ΔΔCt^ Fold change ≤ 2 and P <0.05	Spiked in *cel-miR-39*
Popov, Minkov et al., 2021 [104]	Blood: whole	Yes	Not stated	24:6	8	20:5	7.96	PAXgene blood miRNA isolation kit (PreAnalytiX)	Single miRNA gene examined by qPCR using SYBR Green qPCR Master Mix (Thermo Scientific) on ABI PRISM 7500 system (Applied Biosystems)	2^–ΔΔCt^ P < 0.05	*U6 snRNA*
Popov & Petrov, 2021 [105]	Blood: serum and PBMC	Yes	Yes	30.9	3–11 = range	28 sex matched	Age matched	PAXgene blood miRNA isolation kit (PreAnalytiX)	Single miRNA gene examined by qPCR using SYBR Green qPCR Master Mix (Thermo Scientific) on ABI PRISM 7500 system (Applied Biosystems)	2^–ΔΔCt^	Spiked in *cel-miR-39* for serum *RNAU6-1 (U6)* for PBMC
Ragusa et al., 2020 [81]	Saliva	Yes	Yes	Not stated N = 23	4–8 = range	Not stated N = 12	4–8 = range	miRNeasy kit (Qiagen)	800 miRNAs examined by NanoString nCounter technology	2^–ΔΔCt^	Global Median Normalisation:*miR-21-5p*
				Not Stated N = 53	4–8 = range	Note Stated N = 27	4–8 = range		qPCR of 7 miRNAs by single TaqMan MicroRNA Assays (Applied Biosystems)		
Salloum-Asfar et al., 2021 [73]	Blood: plasma	Yes	Yes	Severe 24:11	8.3 = median	4:4	10.8 = median	miRNeasy kit (Qiagen)	QIAseq miRNA Library kit (Qiagen) Illumina platform on HiSeq 3000/4000 SBS Kit (150 cycles) with targeted depth of 20 million reads per sample	> 50 read counts, absolute fold change > 2 and P < 0.05	Spiked-in *UniSp100*, *UniSp101*, *miR-103*, *miR-191*, *miR-30c*, *miR-451*, *miR-23* and *UniSp6*
				Mild 20:5	8.5 = median						
Sehovic. Et al., 2020 [106]	Saliva	Not stated	Yes	25:14	5	11:14	5.75	mirVana isolation kit	14 miRNA genes by TaqMan microRNA Assay (Applied Biosystems) on Agilent (Stratagene) MX3005P Multiplex qPCR Thermal Cycler	2^–ΔΔCt^ z score > 1.5	*miR-191-5p*
Sell et al., 2020 [107]	Blood: whole	Re-analysis of GEO Dataset GSE67979 (see Huang et al., 2015).							RiboArray miDETECT Array and BioRad qPCR	PCA using QOE	Quantile normalisation / *miR-16-5p*
Shen et al., 2016 [108]	Blood: lymphocyte cells	Not stated	Not stated	Not stated N = 82	Not stated	Not stated N = 64	Not stated	Not stated	Affymetrix Human Genome U133 Plus 2.0 Array mRNA expression data and bioinformatically constructed miRNA-mRNA network	Fold change > 2 and P < 0.05	Quantile normalisation
Vaccaro et al., 2018 [109]	Blood: NOS	Yes	Yes	7:0	7.5	4:0	7.5	TRIzol method	26 miRNA genes by qPCR using SYBR Green qPCR and StepOne System (Applied Biosystems)	2^–ΔΔCt^	geNorm: *miR125a-5p*, *miR181b-5p*, *miR125b-2-3p*, and *miR198*.
Vachev et al., 2013 [110]	Blood	Yes	Not stated	Not stated N = 30	Not stated	Not stated N = 25	Age and sex matched	PAX gene blood miRNA kit (PreAnalytiX)	1898 miRNA genes from miRBase v18.0 using Paraflo miRNA microarray assay (LC Sciences)	Not stated	Not stated
Vasu et al., 2014 [74]	Blood: serum	Not stated	Yes	48:7	11.3	41:14	11.3	miRNeasy kit (Qiagen)	125 miRNA genes using Human Neurological Development & Disease miRNA PCR array (SABiosciences) then qPCR of 14 miRNA genes using SYBR Green on ABI PRISM 7900 Sequence Detection System (Applied Biosystems)	2^–ΔΔCt^ P < 0.05	Spiked in *cel-miR-39*
Yu et al., 2018 [75]	Blood: whole and serum	Yes	Not stated	Not stated N = 2	Aged matched	Not stated N = 3	Aged matched	TRIzol method / mirVana PARIS kit	2,549 miRNA genes from miRBase v21 using Agilent Human miRNA microarrays	2^–ΔΔCt^ P < 0.05 Fold change of 2 fold	*U6 snRNA* and percentage shift method
				18:2	6	20:3	5.5		4 miRNA genes by qPCR on GeneAmp PCR system 9700 (Applied Biological System) with LytCyror 480 II real-time fluorescence PCR (Roche)		
Zamil et al., 2020 [111]	Blood: serum	Yes	Not stated	14:2	6.8	7:9	6.1	miRNeasy kit (Qiagen)	miScript SYBR Green PCR Kit (Qiagen) 40 cycles of qPCR (machine not specified)	2^–ΔΔCt^ P < 0.05 P < 0.01	*U6 snRNA (RNU6-2)*
Zhou et al., 2019 [88]	Blood: NOS	Yes	Yes	14:9	8	Not stated N = 23	Not stated	TRIzol method	9 snRNA gene assay using GenePix microarray and scanner (Axon Instrument)	Custom formula	Not stated

**Table footnote:** Ax = Assessment; DEx = Differential Expression; FDR = False Discovery Rare; NOS = Not otherwise specified; PBMC = Peripheral blood mononuclear cells; PCA using QOE = principal component analysis using Qlucore Omics Explorer—a dynamic, interactive visualization-guided bioinformatics program with a built-in statistical platform; PLS-DA = partial least squared discriminant analysis (weighted sum of absolute regression coefficients); RT = Reverse Transcription; 2^–ΔΔCt^ = 2ˆ(–delta delta CT) method for calculating gene expression. Quantitative polymerase chain reaction assays (qPCR).

**Table 3:** Studies examining ncRNA gene expression in ASD using tissue samples or laboratory methodologies that require complex additional processing or are from deceased persons (N = 17).

Study	Tissue sample	Diagnosis of ASD		Participants				Sample processing		Analysis	
		WHO / DSM criteria	Validated Ax tool	ASD		Control		RNA extraction	ncRNA assay	DEx	Normalisation strategy
				M:F ratio	Mean age	M:F ratio	Mean age				
Abu-Elneel et al., 2008 [112]	Post-mortem cerebellar cortex tissue	Not stated	Not stated	13:0	20.5	13:0	22.6	mirVana miRNA Isolation kit (Ambion)	466 miRNA genes examined by Multiplex qPCR with TaqMan probes on Biosystems 7500HT	2^–ΔΔCt^ P < 0.05	dCHIP software invariant normalisation method
Almehmadi et al., 2020 [79]	Post-mortem dorsolateral prefrontal cortex	Not stated	Not stated	7:0	9.29	7:0	10.4	miRNeasy kit (Qiagen)	2 miRNA gene qPCR using TaqMan RT kit on QuantStudio 12K Flex System (Applied Biosystems)	2^–ΔΔCt^ P < 0.05 P < 0.01	*miR-16*
	Post-mortem amygdala	Not stated	Not stated	8:0	9.88	8:0	11				
Ander et al., 2015 [85]	Post-mortem primary auditory cortex and superior temporal sulcus	Not stated	Yes	5:5	31	6:2	34	RecoverAll Total Nucleic Acid Isolation Kit (Ambion)	5607 small ncRNA genes examined by array (1733 mature miRNAs, 1658 pre-miRNAs and 2216 snoRNAs) on Affymetrix miRNA 3.0 microarrays	Fold change >1.2. P < 0.005	Partek Genomic Suite v6.6 using Robust Multiarray Averaging (RMA)
Bleazard., 2017 [113]	Lymphoblastoid cell lines	Yes	Yes	Not stated N = 42	Not stated	Not stated N = 10	Age matched	miRVana extraction kit (Ambion)	RNA-seq using small RNA library preparation (AB/Life technologies) on SOLiD 4.0 analyzer. Subsequent qPCR using Taqman Fast Advanced Master Mix on Quantstudio 12K Flex (Applied Biosystems)	Read count via Python HTSeq-count package and DESeq2 in R.	3 miRNA genes used for endogenous controls
Frye et al., 2021 [114]	Lymphoblastoid cell lines	Not stated	Yes	10:0	9.6	7:0 (unrelated); 10:0 (sibling)	6.9	TRIzol method miRNeasy kit (Qiagen)	RNA-seq using single end sequencing on Illumina Hiseq4000. Subsequent qPCR using miScript II RT kit (Qiagen) on a QuantStudioTM 6 Flex Real-Time PCR System (Thermo Fisher Scientific)	Log2 fold change P < 0.05 P < 0.01	*cel-miR-39* and *RNU6*. Normalisation of sequence counts (dividing counts by a library size parameter of corresponding sample)
Gandal et al., 2018 [87]	Post-mortem cerebral cortex	Not stated	Not stated	Not stated N = 51	Not stated	Not stated	Not stated	Not stated	Pooled RNA-Seq quantifications data imputed using RSEM guided by Gencode v19 annotations from The PsychENCODE project	Log2FC FDR < 0.05	Not stated
Moore et al., 2019[115]	Neuronal stem cells (Induced pluripotent stem cells derived from dermal fibroblasts)	Not stated	Yes	3:0	26	3.0	58	TRIzol method	Single miRNA gene examined using TaqMan mature miRNA assay (Applied Biosystems)	2^–ΔΔCt^	*U6 snRNA*
Mor et al., 2015 [78]	Post-mortem prefrontal cortex Brodmann’s area	Not stated	Yes	10:2	32	11:1	28.8	miRNeasy kit (Qiagen)	Illumina’s TruSeq Small RNA Sample Prep Kit on Illumina’s MiSeq system. Subsequent qPCR with FastStart Universal SYBR Green Master (Roche) on ABI ViiA 7 system	miRAnalyzer DEx tool	*U6 snRNA* and DSEQ read count normalisation
Nguyen, Lepleux et al., 2016 [71]	Olfactory mucosal cells	Yes	Not stated	Not stated N = 8	Not stated	Not stated N = 6	Not stated	mirVana miRNA isolation kit (Life Technologies) / TRIzol method and miRNeasy kit (Qiagen)	667 miRNA genes from miRBase v10 examined using TaqMan Arrays A and B. Validation of target miRNA genes using SYBR Green Power Mix (Life Technologies) by qPCR-HD-GPC platform (Paris)	2^–ΔΔCt^	*miR-221*
	Primary skin fibroblasts			4:1	5–41 = range	N = 4	Not stated				
	Blood: PBMC			8:1	5–18 = range	N = 20	Not stated				
Nguyen, Fregeac et al., 2018 [82]	Post-mortem temporal lobe	Not stated	Yes	3:2	6	4 sex matched	Age matched	TRIzol method and miRNeasy kit (Qiagen)	RNA-seq using TruSeq Stranded mRNA LT Sample Prep Kit (Illumina) on Illumina HiSeq2500.27 miRNA genes examined using qPCR on the Fluidigm 48.48 chip	Mean of two housekeeping miRNAgenes and average of all controls. Fold change > 1.2 P < 0.001	*Housekeeping miRNA genes*: *miR-106a and miR-17* Geometric mean of *CYC1*, *GPBP1*, *RPL13A*, and *SDHA*
Pagan et al., 2017 [72]	Post-mortem pineal gland	Yes	Yes	8:1	39 = median	20:2	36.5 = median	mirVana PARIS kit (Ambion)	Single miRNA gene examined by qPCR (machine not specified) using miScript reverse transcription kit and SYBR Green PCR kit (Qiagen)	2^–ΔΔCt^ P < 0.0001	Spiked in *cel-miR39*, *cel-miR54* and *cel-miR238* (Qiagen)
	Blood: plasma			n54	Not stated	70	Not stated				
Sarachana et al., 2010 [116]	Lymphoblastoid cell lines	Not stated	Yes	5:0	Not stated	9:0	Not stated	TRIzol method and mirVana miRNA Isolation kit	1237 miRNA genes examined by custom-printed miRNA microarrays from Microarray CORE Facility of the National Human Genome Research Institute. Subsequent qPCR of 4 miRNAs using miRNA TaqMan assays (Applied Biosystems)	Pavlidis template matching analyses P ≤ 0.05	*RNU24* and quantile normalisation
Seno et al., 2010 [117]	Lymphoblastoid cell lines	Not stated	Yes	13:7	Not stated	19.3	Not stated	miRVana miRNA Isolation Kit	Illumina HumanRef-8 V3 gene expression arrays. Subsequent qPCR using SYBR Green (Stratagene) with TaqMan microRNA assay kits (Applied Biosystems)	2^–ΔΔCt^ Fold change ≥ 1.5	*TMEM32* and quantile normalisation
Stamova et al., 2015 [86]	Post-mortem superior temporal sulcus gyrus and primary auditory cortex	Not stated	Not stated	4:4	27	4:2	27	RecoverAll Total Nucleic Acid Isolation Kit	1123 miRNA genes from miRBase v17 and 2,216 snoRNA / scaRNA genes examined by Affymetrix micro-RNA 3.0 microarray	P < .005 Fold-change >1.2	Robust Multichip Averaging (RMA) normalisation via Partek Genomic Suite v6.6
Talebizadeh et al., 2008 [83]	Lymphoblastoid cell lines	Not stated	Yes	3:3	10	3:3	10.5	mirVana miRNA isolation kit	470 miRNA genes from miRBase v9 examined using Paraflo miRNA microarray assay (LC Sciences) and subsequent qPCR with TaqMan assay (Applied Biosystems)	2^–ΔΔCt^ Fold change > 1.5 P < 0.001	*RNU24* (qPCR) Locally-weighted Regression method on the background-subtracted data
Wright et al., 2017 [43]	Post-mortem dorsolateral prefrontal: Brodmann areas 46 and 9	Not stated	Not stated	10:3	22	30:9	22	RNeasy kit (Qiagen)	RNA-seq using TruSeq Stranded Total RNA Library Preparation kit on the HiSeq 2000. Sequencing depth 80–120 million reads per sample	featureCounts FDR < 0.05	Reads Per Kilobase of Gene per Million mapped reads values transformed using log2 = log2(RPKM+1)
Wu et al., 2016 [80]	Post-mortem combined frontal cortex, temporal lobe and cerebellar vermis	Not stated	Yes	45:10	2–81 = range	38:8	8–62 = range	miRNeasy kit (Qiagen)	699 miRNA genes examined following genome wide miRNA expression profiling using Illumina small RNA sequencing. Subsequent qPCR of 10 miRNA genes using miScript RT, Primer Assays kits and SYBR Green PCR Kits (Qiagen) on Roche LightCycler 480 instrument	Log2 fold changes with linear mixed effects model	*RNU6B*

**Table footnote:** Ax = Assessment; DEx = Differential Expression; FDR = False Discovery Rare; PBMC = Peripheral blood mononuclear cells; RT = Reverse Transcription; 2^–ΔΔCt^ = 2ˆ(–delta delta CT) method for calculating gene expression. Quantitative polymerase chain reaction assays (qPCR).

**Table 4:** Overview of individual miRNA genes with differential expression in ASD reported in two or more studies presented by tissue.

miRNA gene	miRBase Accession Number	Blood	Saliva	Brain / neural	LCL (or specified)	Studies
*let-7a-5p*	MIMAT0000062	↓	→	↓		Hicks, Carpenter et al., 2020 [96]; Huang et al., 2015 [98]; Mor et al., 2015 [78];
*let-7b-5p*	MIMAT0000063	↑	↓		↓	Bleazard., 2017 [113]; Ragusa et al., 2020 [81]; Salloum-Asfar et al., 2021 [73]
*miR-7-5p*	MIMAT0000252	↑ ↓	↑ ↑	↑		Hicks, Ignacio et al., 2016 [95]; Jyonouchi et al., 2019 [100]; Mor et al., 2015 [78]; Salloum-Asfar et al., 2021 [73]; Sehovic et al., 2020 [106]
*miR-10a-5p*	MIMAT0000253			↑	↓	Frye et al., 2021 [114]; Wu et al., 2016 [80]
*miR-15a-5p*	MIMAT0000068	↑ ↓				Huang et al., 2015 [98]; Salloum-Asfar et al., 2021 [73]
*miR-16-5p*	MIMAT0000069	↓	↓			Huang et al., 2015 [98]; Ragusa et al., 2020 [81]
*miR-19a-3p*	MIMAT0000073	↑ ↑ ↓		↑		Mor et al., 2015 [78]; Ozkul et al., 2020 [103]; Mor et al., 2015 [78]; Salloum-Asfar et al., 2021 [73]; Yu et al., 2018 [75]
*miR-19b-3p*	MIMAT0000074	↑ ↑ ↓		↑		Cui et al., 2021 [92]; Huang et al., 2015 [98]; Mor et al., (2015) [78]; Vasu et al., 2014 [74]
*miR-20b-5p*	MIMAT0001413	↑		↑	↓	Frye et al., 2021 [114]; Salloum-Asfar et al., 2021 [73]; Wu et al., 2016 [80]
*miR-21-3p*	MIMAT0004494			↑ ↑		Mor et al., 2015 [78]; Wu et al., 2016 [80]
*miR-23a-3p*	MIMAT0000078	↑	↓	↑		Atwan et al., 2020 [90]; Sehovic et al., 2020 [106]; Wu et al., 2016 [80]
*miR-23a-5p*	MIMAT0004496				↑ ↑	Sarachana et al., 2010 [116]; Talebizadeh et al., 2008 [83]
*miR-27a-3p*	MIMAT0000084	↑ ↓	↓ →			Hicks, Ignacio et al., 2016 [95]; Sehovic et al., 2020 [106]; Vaccaro et al., 2018 [109]; Vasu et al., 2014 [74]
*miR-28-3p*	MIMAT0004502	↓	↓			Hicks, Ignacio et al., 2016 [95]; Salloum-Asfar et al., 2021 [73]
*miR-29c-3p*	MIMAT0000681	↑ ↓				Eftekharian et al., 2019 [93]; Salloum-Asfar et al., 2021 [73]
*miR-32-5p*	MIMAT0000090		↓ ↓		↑	Bleazard 2017 [113]; Hicks, Ignacio et al., 2016 [95]; Sehovic et al., 2020 [106]
*miR-34c-5p*	MIMAT0000686	↑			↓	Bleazard., 2017 [113]; Vaccaro et al., 2018 [109]
*miR-99a-5p*	MIMAT0000097	↓ →		↓	↓	Bleazard., 2017 [113]; Salloum-Asfar et al., 2021 [73]; Vaccaro et al., 2018 [109]
*miR-101-3p*	MIMAT0000099	↑ ↑				Salloum-Asfar et al., 2021 [73]; Vasu et al., 2014 [74]
*miR-103a-3p*	MIMAT0000101	↓ ↓				Huang et al., 2015 [98]; Jyonouchi et al., 2019 [100]
*miR-106b-5p*	MIMAT0000680	↑ ↑↑ ↑		→	↓	Nguyen et al., 2016 [71]; Sarachana et al., 2010 [116]; Salloum-Asfar et al., 2021 [73]; Vasu et al., 2014 [74]; Yu et al., 2018 [75]
*miR-107*	MIMAT0000104			↑	↑	Sarachana et al., 2010 [116]; Wu et al., 2016 [80]
*miR-125b-5p*	MIMAT0000423				↑ ↓	Bleazard 2017, Seno et al., 2011
*miR-127-3p*	MIMAT0000446	↓	↑ →			Hicks, Ignacio et al., 2016 [95]; Salloum-Asfar et al., 2021 [73]; Sehovic et al., 2020 [106]
*miR-132-5p*	MIMAT0004594				↑ ↑ ↓	Bleazard 2017 [113]; Sarachana et al., 2010 [116]; Talebizadeh et al., 2008 [83]
*miR-134-5p*	MIMAT0000447	↑ ↓ ↓				Hirsch et al., 2018 [97]; Jyonouchi et al., 2019 [100]; Salloum-Asfar et al., 2021 [73]
*miR-140-3p*	MIMAT0004597	↑ →	↑ ↓			Cirnigliaro et al., 2017 [91]; Hicks, Ignacio et al., 2016 [95]; Sehovic et al., 2020 [106]; Yu et al., 2018 [75]
*mir-141-3p*	MIMAT0000432	↑ ↑	↑			Ragusa et al., 2020 [81]; Salloum-Asfar et al., 2021 [73]
*miR-144-3p*	MIMAT0000436	↑ ↓		↑ ↑		Mor et al., 2015 [78]; Nakata et al., 2019 [102]; Salloum-Asfar et al., 2021 [73]
*miR-146a-5p*	MIMAT0000449	→	↑	↑ ↑ ↑	FB; LCL ↑↑ ↑	Mor et al., 2015 [78]; Nguyen, Lepleux et al., 2016 [71]; Nguyen, Fregeac et al., 2018 [82]; Ragusa et al., 2020 [81]; Talebizadeh et al., 2008 [83]
*miR-151a-3p*	MIMAT0000757	↓	↓			Hicks, Carpenter et al., 2020 [96]; Vasu et al., 2014 [74]
*miR-155-5p*	MIMAT0000646			↑ ↑↑ ↑ →		Almehmadi et al., 2020 [79]; Mor et al., 2015 [78]; Wu et al., 2016 [80]
*miR-181b-5p*	MIMAT0000257	↓ ↓ →→				Atwan et al., 2020 [90]; Cui et al., 2021 [92]; Vaccaro et al., 2018 [109]; Vasu et al., 2014 [74]
*miR-188-5p*	MIMAT0000457	↑			↑	Bleazard 2017 [113]; Yu et al., 2018 [75]
*miR-193a-5p*	MIMAT0004614	↓ ↓	→			Hicks, Carpenter et al., 2020 [96]; Jyonouchi et al., 2019 [100]; Vaccaro et al., 2018 [109]
*miR-193b-5p*	MIMAT0004767	↑ ↓				Jyonouchi et al., 2019 [100]; Salloum-Asfar et al., 2021 [73]
*miR-195-5p*	MIMAT0000461	↑ ↓				Huang et al., 2015 [98]; Vasu et al., 2014 [74]
*miR-197-5p*	MIMAT0022691	↓ ↓				Kichukova, Popov et al., 2017 [76]; Kichukova, Petrov et al., 2021 [101]
*miR-199b-5p*	MIMAT0000263	↑ ↑			↑	Salloum-Asfar et al., 2021 [73]; Seno et al., 2011 [117]
*miR-219-5p*	MIMAT0000276 or MIMAT0019747			↑	↑	Mor et al., 2015 [78]; Sarachana et al., 2010 [116]
*miR-221-3p*	MIMAT0000278	↓ →		↑		Salloum-Asfar et al., 2021 [73]; Vaccaro et al., 2018 [109]; Wu et al., 2016 [80]
*miR-223-3p*	MIMAT0000280			↑ ↑	↑	Bleazard 2017 [113]; Wu et al., 2016 [80]
*miR-328-3p*	MIMAT0000752	↑ ↓ ↓ ↓				Kichukova, Popov et al., 2017 [76]; Nakata et al., 2019 [102]; Popov, Minchev et al., 2018 [77]; Salloum-Asfar et al., 2021 [73]
*miR-335-3p*	MIMAT0004703		↑↑ →	↑		Hicks, Ignacio et al., 2016 [95]; Sehovic et al., 2020 [106]; Wu et al., 2016 [80]
*miR-363-3p*	MIMAT0000707			↑	↓	Frye et al., 2021 [114]; Wu et al., 2016 [80]
*miR-379-5p*	MIMAT0000733	↓		↑		Jyonouchi et al., 2019 [100]; Mor et al., 2015 [78]
*miR-424-5p*	MIMAT0001341	↑ ↓		↑		Kichukova, Popov et al., 2017 [76]; Kichukova, Petrov et al., 2021 [101]; Wu et al., 2016 [80]
*miR-451a*	MIMAT0001631	↓ ↑	↓	↑ ↑	↓	Frye et al., 2021 [114]; Huang et al., 2015 [98]; Mor et al., 2015 [78]; Pagan et al., 2017 [72]; Ragusa et al., 2020 [81]
*miR-486-3p*	MIMAT0004762	↑ ↓			↑	Popov, Minkov et al., 2021 [104]; Seno et al., 2011 [117]; Yu et al., 2018 [75]
*miR-494-3p*	MIMAT0002816	↑		↑		Huang et al., 2015 [98]; Mor et al., 2015 [78]
*miR-500a-5p*	MIMAT0004773	↓ ↓				Kichukova, Popov et al., 2017 [76]; Kichukova, Petrov et al., 2021 [101]
*miR-574-3p*	MIMAT0003239	↓ ↓				Huang et al., 2015 [98]; Jyonouchi et al., 2019 [100]
*miR-619a-5p*	MIMAT0026622	↑ ↑		↓		Kichukova, Popov et al., 2017 [76]; Popov & Petrov 2021 [105]; Wu et al., 2016 [80]
*miR-664a-3p*	MIMAT0005949	↑ ↑		↑		Ander et al., 2015 [85]; Kichukova, Popov et al., 2017 [76]; Kichukova, Petrov et al., 2021 [101]
*miR-874-3p*	MIMAT0004911	↑ ↓		↑		Nakata et al., 2019 [102]; Salloum-Asfar et al., 2021 [73]; Wu et al., 2016 [80]
*miR-940*	MIMAT0004983	↓		↑		Huang et al., 2015 [98]; Wu et al., 2016 [80]
*miR-3135a*	MIMAT0015001	↓ ↓				Kichukova, Popov et al., 2017 [76]; Popov, Minchev et al., 2018 [77]
*miR-3613-3p*	MIMAT0017991	↓ ↓				Huang et al., 2015 [98]; Ozkul et al., 2020 [103]
*miR-3613-5p*	MIMAT0017990	↑			↑	Bleazard 2017 [113]; Salloum-Asfar et al 2021 [73]
*miR-4270*	MIMAT0016900	↑ ↑				Huang et al 2015 [98]; Sell et al 2020 [107]
*miR-4728-5p*	MIMAT0019849	↑ ↑				Huang et al 2015 [98]; Yu et al 2018 [75]
*miR-4732-5p*	MIMAT0019855	↑ ↑				Jyonouchi et al 2019 [100]; Salloum-Asfar et al., 2021 [73]
*miR-4742-3p*	MIMAT0019873	↑		↓		Ander et al., 2015 [85]; Salloum-Asfar et al., 2021 [73]
*miR-6086*	MIMAT0023711	↑ ↑				Huang et al., 2015 [98]; Yu et al., 2018 [75]

**Abbreviations used:** FB = primary skin fibroblasts; LCL = Lymphoblastoid cell line; ↑ = increased expression

↑ = decreased expression; → = non-significant expression change (*where reported*).

**Table 5:** Studies examining differential expression of ncRNA classes other than miRNA in ASD.

Study	Details	Tissue	Increased expression	Decreased expression
Ander et al., (2015) [85]	ncRNA expression profiles	Superior Temporal Sulcus	*SNORA11C*, *SNORA27*, *SNORA71E* (referred to as *ACA39*)	*SNORD13P1* (referred to as U13 paralogue on Chromosome 1)
		Primary Auditory Cortex	-	*SNORA22*, *SCARNA6*, *SNORD13P2* (referred to as U13 paralogue on Chromosome 2), *SNORD13P3* (referred to as U13 paralogue on Chromosome 3), (U13 paralogue on chromosome 11—no corresponding HGNC gene symbol)
Cheng et al., (2020) [84]	20 ncRNA gene diagnostic signature blood test	Peripheral blood sample	ncRNA genes with "discrepancy between ASD and control samples", but expression data not reported. The model included 6 ncRNA genes eligible for systemic review inclusion: • One snoRNA gene: *RNU105B* • One Y RNA pseudogene: *RNY1P11* • Five snRNA genes: *RNU1-16P*, *RNU6-258P*, *RNU6-485P*, *RNU6-549P and RNVU1-15*	
Gandal et al., (2018) [87]	PsychENCODE Consortium study. Transcriptome-wide isoform-level data from 52 individuals with ASD	Post-mortem brain samples	1363 ncRNA genes annotated from RNA-seq data (novel and known in ensemble using genecodeV27 nomenclature). 178 ncRNA genes differentially expressed in ASD (vast majority not HGNC approved shorter classes–most abundant class are 60 lincRNA genes). Only one apparent HGNC approved shorter ncRNA gene identified (snoRNA gene–see below)	
			-	*SNORD-3B-2*
Salloum-Asfar et al., (2021) [73]	ncRNA expression profiles	Peripheral blood plasma	*piR-hsa-1282*, *piR-hsa-12790*, *piR-hsa-23326*, *piR-hsa-1207*, *piR-hsa-28131*, *piR-hsa-6463*, *piR-hsa-1242*, *piR-hsa-27493*, *piR-hsa-27620*, *piR-hsa-27621*, *piR-hsa-27140*, *piR-hsa-1243*, *piR-hsa-23533*, *piR-hsa-23248*, *piR-hsa-28876*, *piR-hsa-27622*, *piR-hsa-1177*, *piR-hsa-28190*, *piR-hsa-5937*, *piR-hsa-24672*, *piR-hsa-28877* *SNORD3C*, *SNORD69*, *SNORD51*, *SNORD10*, *SNORD22*, *SNORD24*, *SNORD102*, *SNORD3A*, *SNORD26*	*piR-hsa-28390*, *piR-hsa-32235*, *piR-hsa-28374*, *piR-hsa-32195*, *piR-hsa-23210*, *piR-hsa-32238*, *piR-hsa-27731*, *piR-hsa-27730*, *piR-hsa-325*, *piR-hsa-27729*, *piR-hsa-1849*, *piR-hsa-32158*, *piR-hsa-23209*, *piR-hsa-32182*, *piR-hsa-32167*, *piR-hsa-32159* *RNY4P36*, *RNY4P6*, *RNY4*, *RNY4P25*, *RNY4P18* *SNORA63B*, *SNORD65*, *SNORA51*, *SNORD57*, *MT-ND1*, *MT-RNR1*, *MT-ND5*, *MT-ND3*, *MT-TW*, *MT-TE*, *MT-RNR2*, *MT-TS2*, *MT-TS1*, *MT-TG*, *MT-TT*, *MT-TV*, *MT-TH*, *MT-CO3*, *MT-TK*, *MT-TI*, *MT-TP*, *MT-TQ*, *MT-TY*, *MT-TN*, *MT-TL2*, *MT-TL1*, *MT-TD*, *MT-TC*, *MT-TR*
Stamova et al., (2015) [86]	Assessment of brain region and age-related ‘small’ ncRNA expression patterns	Post-mortem Superior Temporal Sulcus Association Cortex versus Primary Auditory Cortex	*SNORD114-14* (referred to as *snoRNA 14qII-14*), *SNORD88* (referred to by ensembl ID: ENSG00000221611), (‘*snoU13*’ gene on chromosome 2 referred to by ensembl ID ENSG00000239170 was also reported, but has no approved HGNC ncRNA gene symbol)	*SNORA71D* (referred to as *U71d*)
		Expression of ncRNA with increasing age in Superior Temporal Sulcus	*SNORA73A* (referred to as *U17a*), *SNORA22B* (referred to by ensembl ID ENSG00000206603), *SNORA48* (referred to by ensembl ID ENSG00000212445), (‘*snoU13*’ gene on chromosome 9 referred to by ensembl ID ENSG00000239055 was also reported, but has no approved HGNC ncRNA gene symbol)	*SCARNA12* (referred to as *U89*)
Wright et al., (2017) [43]	Genome wide differential expression analysis	Post-mortem dorsolateral prefrontal cortex	*SNORA54*	*SNORA74A*, *SNORA53*, *SNORD17*, *SNORA74B*, *SNORD114-23*
Zhou et al., (2019) [88]	9 snRNA diagnostic signature from same datasets as Cheng et al., 2020 [84], with 4 additional snRNAs.	Peripheral blood sample	ncRNA genes with "discrepancy between ASD and control samples", but expression data not reported. snRNA diagnostic signature model included 9 eligible snRNA genes: • 5 snRNA genes overlapping with Cheng et al., (2020) [84] model: *RNU1-16P*, *RNU6-258P*, *RNU6-485P*, *RNU6-549P* and *RNVU1-15* • 4 additional snRNA genes: *RNU6-1031P*, *RNU6-335P*, *RNU6-98P* and *RNU6ATAC26P*	

### Non-coding RNA with differential expression in ASD

The systematic review revealed 64 miRNA genes with differential expression in more than one study (Table 4). Twenty-nine of these miRNA genes had differential expression in opposing directions. Four miRNA genes had differential expression in the same direction in the same tissue type in at least 3 separate studies. These were in bloods samples for *miR-106b-5p* [73–75] and *miR-328-3p* [73, 76, 77], which had increased and decreased expression, respectively. The other miRNA gene was *miR-155-5p* which had increased expression in post-mortem brain samples [78–80]. Finally, *miR-146a-5p* had consistent, increased differential expression across several different tissue types as reported in four studies [71, 81–83]. These were from saliva, primary skin fibroblasts, lymphoblastoid cell lines, olfactory mucosal cells and post-mortem brain samples from the pre-fontal cortex and temporal lobe, respectively. Seven research studies examined ncRNA gene expression, other than miRNA, in association with ASD (Table 5). From these studies, differential expression was reported in individual genes from ncRNA classes including: snoRNA [43, 73, 84–87], snRNA [84, 88], piRNA [73] and Y RNA genes [73]. Differential expression of one or more snoRNA genes was reported by six studies, but no individual snoRNA gene or other individual ncRNA genes (excluding miRNA genes) had differential expression reported in more than one study.

### Data synthesis and meta-analysis

Functional enrichment analysis using DIANA-miRPath v3.0 online interface [65] was performed with interrogation of the four key miRNA genes identified in this review (*miR-106b-5p*, *miR-328-3p*, *miR-146a-5p* and *miR-155-5p*) versus Gene Ontology (GO) categories. Clustering with the highest enrichment significance levels were seen in ‘ion binding’ and ‘organelle function’ GO categories, which can be visualised within the heatmap generated (S1 Fig). For the planned meta-analysis, we extracted all available ncRNA expression data from each study. Data for most studies was incomplete for meta-analysis, therefore we contacted corresponding authors, but were only able to obtain raw datasets from a small number of studies. Considering all included studies, a range of data elements were used to capture ncRNA gene differential expression. Only 17 papers included both fold change and associated statistical findings [43, 73, 75, 77, 78, 80–82, 85, 90, 101–103, 110, 113, 114, 117] and often the latter was not corrected for multiple testing. The different data types, levels of data processing and in many instances inaccessible data, made meta-analysis unsuitable [118]. Many papers only reported p-values for miRNAs that were found to be differentially expressed (i.e. did not report those with non-significant expression). Although methods to combine p-values have been proposed [119], we found that the use of different statistical tests, different hypotheses (one-sided or two-sided) and any adjustment for multiple comparisons often being unknown, made this inappropriate. Originally plans were made for a series of statistical and publication bias analytical assessments as part of a meta-analysis, but these were not possible [64, 68, 69]. We added a field into the quality assessment related to statistical analysis given the complexities of analysing complex data sets and multiple testing in the included studies [120]. The quality assessment using adapted QUADAS-2 [70] is shown in S1 Table.

## Discussion

We consider here our findings from 46 studies that examined miRNA gene expression and 7 studies examining other classes of ncRNA.

### Differential expression of miRNA genes in ASD

Several miRNA genes have been reported to have differential expression in two or more studies (Table 4). Whilst this initially appears promising, many of these are in opposing directions. This may relate to tissue specificity of miRNA gene expression [71], type I errors related to high numbers of miRNA genes tested and/or statistical tests being performed [121] or reflect the heterogenous nature of ASD aetiology or the study populations examined [15, 122]. Further issues around study quality and bias are considered later in the discussion. Only four miRNAs had differential expression in the same direction and tissue type in at least three studies: *miR-106b-5p*, *miR-146a-5p*, *miR-155-5p* and *miR-328-3p*. Intriguingly, in addition to the studies in our systematic review, a further single case study examining genome-wide differential miRNA gene expression from the post-mortem prefrontal cortex of a single deceased individual with ASD compared with a non-ASD sibling control without ASD (i.e. ASD of N = 1) also found *miR-106b-5p* and *miR-146a-5p* were in their top six differentially expressed miRNA genes [123]. It is instructive to consider the 4 notable miRNA genes identified in our systematic review in more detail, although caution should be exercised given the possibility of selective research and/or reporting and high levels of potential bias found from our quality assessments.

### Four notable miRNA genes with differential expression in ASD

#### miR-106b-5p

It has previously been reported that *miR-106b-5p* has altered expression in schizophrenia [124]. The finding that ASD and childhood onset schizophrenia both share altered expression is under research scrutiny [125], although both these groups also have high associated rates of pathogenic copy number variants and brain trauma [126] and there is a long history of some diagnostic overlap [127]. *miR-106b-5p* has a wide influence on various biological processes including cancer [128, 129] and in isolation is unlikely to demonstrate disease specificity.

#### miR-146a-5

*miR-146a-5p* was found to have uniformly increased expression in our systematic review across a wide range of tissue types including saliva, primary skin fibroblasts, lymphoblastoid cell line, olfactory mucosal cells and post-mortem brain samples from the prefrontal cortex and temporal lobe, respectively [71, 78, 81–83]. One of these studies examined tissue and disease specificity of *miR-146a-5p* (with three other miRNA genes) and found no differential expression in peripheral blood mononuclear cells (PBMC) from a group of ASD patients compared to controls [71]. *miR-146a-5p* has also been implicated in a number of biological processes including regulation of the development of viral infections [130] and cancer tumour suppression [131], for example in the inhibition of both EGFR and NF-kB signalling and reduction of the metastatic potential of cancers [132].

#### miR-155-5p

*miR-155-5p* showed a degree of uniformity in our systematic review, with increased expression in the amygdala, prefrontal cortex and temporal cortex regions in three post-mortem studies [78–80] but with no significant differential expression found in dorsolateral prefrontal cortex [79]. *miR-155-5p* has been implicated in inflammatory processes [133, 134] and the modulation of cancer [135]. *miR-155-5p* expression appears to be involved in impaired development of dendritic cells, B cells and T cells and is important for immune response [136, 137]. Moreover, it was one of several differentially expressed miRNA genes associated with a basket of neurodegenerative diseases, including idiopathic Parkinson’s disease, where *miR-155-5p* has been reported to have increased expression [138].

#### miR-328-3p

In our systematic review, *miR-328-3p* was found to have decreased expression in peripheral blood samples in three studies examining serum [76, 77] and plasma [73], respectively [73, 76, 77] but a further study reported increased expression in peripheral blood [102]. *miR-328-3p* has been thought to have a role in cancer, whereby suppression is believed to impair stem cell function, a mechanism hypothesised to prevent ovarian cancer metastasis [139].

#### Functional enrichment analysis

The output of functional enrichment analysis by DIANA-miRPath v3.0 [65] with the four key miRNA genes identified in this systematic review (*miR-106b-5p*, *miR-146a-5p*, *miR-155-5p* and *miR-328-3p*) versus gene ontology categories identified the most significant levels of enrichment in ‘ion binding’ and ‘organelle function’ GO categories (S1 Fig). Ion binding is an interesting finding, given the theories of channelopathy dysregulation in the pathogenesis of ASD [140–143]. However, there are well articulated concerns related to the cautious interpretation of functional enrichment and pathway analysis of miRNA that have been raised within the miRNA research community [144–147]. For example, there have been suggestions that the results from standard analyses are biased by over-represented terms and may suffer from ascertainment bias for the most studied molecular pathways and be limited by selective coverage of annotated genes within a gene set [144]. Some solutions to these challenges have been proposed [144, 145, 147] but are beyond the scope of this review.

### Other ncRNA with differential expression in ASD

Whilst the majority of papers identified in this systematic review examined miRNA gene expression, other ncRNA genes with differential expression were reported in seven papers including differential expression of snoRNA [43, 73, 84–86], snRNA [84, 88], piRNA [73] and Y RNA genes [73] (Table 5). One of these studies Salloum-Asfar and colleagues (2021) [73] was the first to report stable expression of piRNA, snoRNA, Y RNA and tRNA genes in plasma, a helpful attribute for further research. Two of the seven papers were published by the same research group [84, 88], and described overlapping ASD ncRNA ‘diagnostic signatures’ that derived from re-annotation and analysis of expression data from an external dataset with validation using recruited participants. Together these two studies described nine snRNA genes [84], one snoRNA gene [88] and one Y RNA pseudogene in overlapping ncRNA expression diagnostic models measured in blood (Table 5). Unfortunately the corresponding raw data, strength and direction of expression change, and how each ncRNA gene contributed to their ‘signature formula’ models were not clearly reported [84, 88]. The small number of ncRNA gene expression studies in cohorts of individuals with a diagnosis of ASD is in itself an important finding to report, to help shape future research directions, given their cellular mechanisms and theoretical links with ASD. Each ncRNA class with reports of differential expression in ASD found in our review, have been discussed further, in turn.

#### Small nucleolar RNA

Six studies examined differential expression in snoRNA genes in ASD. snoRNA can be divided into three major classes: C/D box snoRNAs (SNORDs), H/ACA box snoRNAs (SNORAs) and small Cajal body‐specific RNAs (scaRNAs) (Table 1). snoRNAs accumulate in the nucleoli of the cell and have roles in post-transcriptional modification and maturation of ribosomal RNA and snRNA [56, 85, 148, 149] and roles in mRNA processing and splicing [150]. There is interest in snoRNA splicing disruption affecting neuronal development and function [151–153]. snoRNA have been associated with a range of human diseases [154] including ASD, and are gathering interest [43, 73, 84–86]. Differentially methylated genomic regions of paternal sperm samples have been associated with ASD-related phenotype at 12 months of age [22]. The paternal sperm genomic loci region exhibiting differential methylation in this study contains fifteen snoRNA genes within the *SNORD-115* cluster, which lies within the Prader-Willi syndrome critical region on chromosome 15. Prader-Willi syndrome is an imprinting condition that can manifest with a neurobehavioral phenotype with aspects of ASD symptomatology [155].

#### Small nuclear RNA

Most snRNA are involved in the major and minor spliceosome complex to splice the introns from pre-messenger RNA [53]. snRNA and the related core spliceosomal U-snRNP complexes are associated with numerous diseases including those with neurological manifestations such as spinal muscular atrophy (SMA), amyotrophic lateral sclerosis and Burn‐McKeown syndrome [156–159]. Some authors have proposed an association of with snRNA with ASD [84, 88, 160], including Zhou and colleagues (2019), identified in this review, who report an ASD-ncRNA ‘diagnostic signature’ in blood comprising entirely of snRNA genes [88].

#### Piwi-interacting RNA

piRNA are frequently considered with miRNA, given their comparable size, and overlapping molecular functions [51]. In contrast to miRNA, piRNA are predominantly expressed in germline cells and function to silence transposable elements and regulate gene expression through RNA cleavage and methylation mechanisms. The role of piRNA is increasingly being described in somatic cells, such as in the nervous system and they have been implicated in neurodevelopmental and neurodegenerative disorders [161]. Rett syndrome is an X-linked dominant neurodevelopmental condition affecting females caused by pathogenic variants in the *MECP2* gene [162]. Rett Syndrome is characterised by developmental regression following a period of apparently normal development, an ASD neurobehavioural phenotype and repetitive hand movements. The *MECP2* gene is responsible for binding to methylated genomic DNA and has epigenetic functions required for neuronal development [163]. Interestingly, *MECP2* knockout mice have increased piRNA expression profiles in the cerebellum [164]. *MECP2* also has roles related to miRNA biogenesis, miRNA binding and lncRNA interactions [163].

#### Y RNA

One study identified in this systematic review reported five Y RNA genes (*RNY4P36*, *RNY4P6*, *RNY4*, *RNY4P25* and *RNY4P18*) with decreased plasma expression in ASD compared with controls [73]. The same study reported four other Y RNA genes with differential expression associated with ‘more symptoms’ of ASD, with increased expression of *RNY4P29* and decreased expression *of RNY3P1*, *RNY3 and RNY4P28*, respectively. Whilst there were no other studies reporting Y RNAs, Cheng and colleagues (2020) included a single Y RNA pseudogene known as *RNY1P11* within their ASD ncRNA diagnostic signature in blood [88], but had no HGNC approved Y RNA genes within their model. Y RNAs were first discovered in the serum of people with systemic lupus erythematosus (SLE), a multisystemic autoimmune condition that can involve the brain [165]. Y RNA have cellular roles related to DNA replication, RNA stability and cellular stress responses [59, 60].

#### Other classes of ncRNA lacking ASD differential expression evidence

Whilst no differential expression findings were forthcoming from this systematic review in relation to vtRNA, tRNA and snaR, we have highlighted some interesting literature relevant to ASD, worthy of further discussion.

#### Vault RNA

vtRNA plays a role in neuronal synapse formation and so are of interest in ASD given postulated aetiologies such as altered neurone development including synapse formation [166]. vtRNA bind to and activate a mitogen-activated protein kinase (MEK) to amplify the RAS-MAPK signalling pathway [167]. There is emerging evidence associating RASopathies (a group of inherited disorders caused by pathogenic variants of genes encoding regulatory proteins within the RAS-MAPK signalling pathway) with an increased prevalence of ASD [168]. One such RASopathy is Legius syndrome, which interestingly has a murine model where the ASD-like neurobehavioral phenotype is ameliorated by MEK inhibitors [169]. Further work related to vtRNA expression in ASD could complement this research to support the possible clinical translation of ASD-related MEK inhibitor drug therapy [169].

#### Transfer RNA

tRNA genes are encoded for by both nuclear and mitochondrial genomes. The mitochondrial genome has been proposed as a genetic modifier for ASD [170] and theories related to mitochondrial dysfunction in ASD have been hypothesised [171]. The mitochondrial genome encodes 22 transfer RNA genes and harbours the majority of pathogenic variants that result in broad and disparate disorders [172]. One report demonstrated a mitochondrial tRNA variant within a single family that was attributed as causative for a heterogeneous group of neurological disorders where ASD was a feature [173].

#### Small NF90 (ILF3) associated RNA

snaR gene expression may also be worthy of further examination in ASD, given their abundant expression within the testis and discrete regions of the brain [52]. Evidence from meta-analysis reports that advanced paternal age as a risk factor for ASD [174], which may be related to increased rates of genomic and epigenomic abnormalities within the germline cells [175]. It is also interesting that polymorphisms of *SNAR-I* (one of twenty snaR genes), is associated with increased lateral ventricle volume [176], which is one of two neuroimaging distinguishing features (alongside increased Pallidum volume) found in a large ASD cohort that underwent high-resolution structural brain scans [177].

### Limitations and quality assessments of studies

#### Quality of data and reporting

There are several limitations that need to be taken seriously both in interpreting the results from this systematic review and in planning for future research. The exact number of ASD participants from all included studies was difficult to ascertain as certain studies were not explicit in descriptions of study populations, and there were occasions where it was difficult to exclude some study population overlap [77, 104, 178]. The use of external datasets and biobank sample resources also made this challenging, with some instances where the same Gene Expression Omnibus (GEO) dataset was used (for example GSE18123 in three studies) [84, 88, 94]. Two of these studies were from one research group that also appeared to use the same internal datasets in both of their studies, but this was not readily apparent in their described methodologies [84, 88]. Most studies use small sample sizes and several studies do not report how the diagnosis of ASD was established (e.g., whether they used validated measures). We identified studies that included participants with ASD present alongside confounding phenotypes for example, individuals with ‘high-functioning’ ASD [102], those who recruited both ASD and control participants from an allergy/immunology clinic [99], and individuals with high levels of consanguinity, epilepsy and dysmorphism [112], that may influence miRNA expression [179–181]. Participants were frequently recruited from convenience samples or clinic populations and many studies had a limited description of control groups with few or no assessments to characterise phenotype variations. These factors are further challenged by the heterogeneity of ASD and the use of small sample sizes [15, 182]. We also recognised a large variation in the methods used to determine ncRNA gene expression and many studies omit important methodological details related to these.

#### Meta-analysis and data synthesis

Statistical methodological quality in the studies are highly variable with many instances of small sample sizes and studies using inappropriate statistical tests. It is unclear in some studies whether correction for multiple testing has been applied and, where stated, different methods have been used such as Bonferroni or Benjamini-Hochberg correction. For meta-analysis, we considered methods to combine p-values [119], such as Stouffer’s method [183] that is generally preferred when different weights are attributed to the p-values being combined. However, it is not clear how the direction of differential expression (often presented as fold change) should be incorporated. Some authors recommend the removal of genes with conflicting differential expression, so that only the genes with the same fold change are combined [184] and others suggest that one-sided p-values can be used to take the direction of fold-change into account. When not specified, the p-values given are presumably two-sided but one-sided p-values are sometimes reported. We also observed different statistical tests, including t-tests, Mann-Whitney U-tests and Tukey’s multiple comparison tests to provide the p-values. These were often reported as simple inequalities rather than precise values, making it unlikely that useful information could be extracted from their combination. High degrees of heterogeneity were apparent across studies with respect to participants, sample types and expression assays. It is well recognised that different cell types have tissue specific ‘miRNomes’ and comparing this ncRNA expression data therefore might not be appropriate [185]. Despite contacting several authors, we were not able to obtain full data sets in several cases. In summary, our planned strategy for meta-analysis and integration of the findings from different studies was not possible [118] because of the large variation in data presentation, availability, statistical analysis used and many instances of poor reporting.

#### Factors affecting ncRNA gene expression

The field of ncRNA gene expression studies is littered with challenges in the interpretation of findings. Disease or developmental states may not be the only factors altering ncRNA expression. Exercise [186], sleep [187], nutritional intake [188, 189] and infection [190] are just some factors that may impact ncRNA expression. Interestingly, sleep [191], nutrition [192], bowel habit [193] and exercise [194] may be markedly different in people with ASD compared to neurotypical people, raising the prospect that ncRNA differential expression findings may be as a result of ASD and its patterns, lifestyles and associations rather than (or as well as) aetiological. This is currently unclear and so research methodologies should attempt to examine and control for this where possible. There are numerous ways that ncRNAs are deployed in biological processes. As in multifactorial models of ASD aetiology [195], the role of ncRNAs may also be multifaceted and interactive.

We also know that sample collection, RNA extraction, purification, storage, handling, and testing conditions can greatly impact ncRNA expression [196–198]. For example, the use of an EDTA anticoagulant appears to influence specific miRNA expression, particularly after longer EDTA exposure times [196]. In our systematic review, EDTA blood tubes were used in several studies [73, 75, 76, 89, 90, 93, 101, 105] with only a few studies using PAXgene blood RNA tubes [94, 102, 104, 110] and many studies omitted details about blood sample collection, including anticoagulant exposure timings. Quantity and quality of centrifuging in blood has also been shown to alter the proportion of intra and extracellular components that may demonstrate different miRNA expression properties [197]. Challenges related to ncRNA data normalisation approaches also support the need for standardisation [199]. Caution is also required for the interpretation of post-mortem samples. In life, hypoxia is known to change miRNA function and expression [200] and so it is not surprising that post-mortem miRNAs are altered through the process of death with degradation happening in different ways at different rates [201, 202]. Post-mortem ncRNA gene expression studies therefore need to include supplementary tests to explore degradation to aid interpretation. In summary, the process of measuring ncRNA gene expression requires quality control and clear detailed reporting to allow comparison between studies for meaningful interpretation.

#### Differential gene expression in opposing directions

Our review findings of studies reporting miRNA genes with differential expression in opposite directions needs further consideration. Another systematic review in type two diabetes mellitus reported that two thirds of differentially expressed miRNA genes were found in opposite directions [63]. Whilst this may suggest poor methodologies or reporting bias we should be cautious about how we interpret this. Some miRNA genes appear to have greater tissue specificity than others [203]. In the context of cancer, opposing directions of miRNA differential gene expression in *miR-125b* is thought to represent oncogenic characteristics when expression is increased and loss of tumour suppressive functions when expression is decreased [204]. Differential expression in opposing directions of individual miRNA genes was observed in this systematic review on a population level, but also on an individual level [112]. There is evidence that direction of miRNA (and other ncRNA) differential expression may change with age [86] or over time and may respond to environmental exposures such as smoking [205] and alcohol [206]. Whilst numerous miRNA genes have been associated with neurodevelopmental or neurodegenerative diseases [138] there is still much work to be done to understand whether miRNA differential expression may play a role in aetiology or to the numerous other factors described above including a response to the condition itself.

#### Expression assays for ncRNA

Various technologies for measuring ncRNA expression levels have been used in the studies, each with different strengths and limitations [207]. Quantitative polymerase chain reaction assays (qPCR) are based on the amplification of target ncRNA genes of known sequence. Although qPCR assays are known for their high sensitivity and specificity, the sensitivity does depend on the target abundance and the efficiency of the amplification [208]. If there are closely related sequences to the target sequence, there is a risk of false amplification. The many different protocols, reagents, and analysis methods and lack of technical information led to recommendations for qPCR assay design and data reporting, or “minimum information for the publication of qPCR experiments” (MIQE) [209]. qPCR assays can be expensive as each target requires specific primers and probes and they are commonly used to validate gene expression changes identified by other methods, such as microarrays or Next-Generation Sequencing (NGS). Microarrays are cost-effective and have been widely used in ncRNA gene expression research. However, they may not be sensitive enough to detect expression of low-abundance ncRNA genes and can suffer from dynamic range issues which affect the quantification of highly abundant transcripts [210]. Microarray results can also be influenced by probe design bias, as the performance of the probes may vary depending on their sequence. Differences in hybridisation as well as normalisation issues mean that RNA sequencing is sometimes preferred [211]. NGS has revolutionised ncRNA research by allowing comprehensive profiling of ncRNAs. However, biases in library preparation methods, including at ligation, reverse transcription, and amplification steps, and sequencing errors, can all affect the accuracy of ncRNA identification and quantification [212]. Furthermore, NGS generates huge amounts of data, requiring advanced bioinformatics tools and computational resources for data analysis.

#### Implications for clinical practice

At the current time there are no implications for clinical practice that we could reliably draw from these results, with limited evidence to support ncRNA gene expression as biomarkers for ASD. The ncRNA genes with differential expression identified in this systematic review have all been implicated in several other diseases and biological processes and there is limited or no reporting of any high sensitivity and/or specificity scores or validation studies. There are also limited descriptions of phenotypes in the ASD groups. There is, however, enough promise to suggest that continuing to research in this field has potential to improve our understanding of mechanisms associated with neurodevelopmental differences such as ASD.

#### Implications for research

By contrast there are many implications for research to consider. The finding that there is limited research examining gene expression in classes of ncRNA other than miRNA is important to report. This shines a light on the omission in the research literature. Given that miRNA gene silencing occurs in many tissue types including in the developing brain [213], it is intriguing that four proteins critical for miRNA biogenesis [214] are encoded by genes associated with Mendelian disorders where ASD and overlapping neurobehavioral phenotypes are highly prevalent: *DRCG8* (included within the deleted region in chromosome 22q11.2) [215], *MECP2* (Rett syndrome) [216], *FOXG1* [217] and *FMR1* (Fragile X) [218, 219]. As key regulators of gene expression, miRNA may have a role in modifying genetic variants demonstrating incomplete penetrance and variable expressivity [220]. This theory is interesting, considering the multiple examples of recurrent pathogenic CNVs associated with variable ASD risk [19].

Some standardisation is required to overcome the large variability in quality and reporting of ncRNA gene expression in ASD. Improved methodologies and reporting would greatly benefit the research endeavour. Alongside MIQE mentioned above, we recommend researchers work to the FAIR Guiding Principles for scientific data management and stewardship (2016) [221] to improve the findability, accessibility, interoperability, and reusability of ncRNA expression data in ASD and other ncRNA expression studies. This would provide the standardisation and authentication necessary for data to be reusable. Feature level extraction output (FLEO) files have been recommended as published gene lists (PGL data) and gene expression data matrices (GEDMs) have been deemed unsuitable for meta-analysis due to their dependence on the pre-processing used [118]. Sharing research data between research groups comes with challenges [222] and public sharing of raw data in biomedical microarray studies appears to be more likely for studies published in high impact journals and when lead authors are more experienced researchers [223, 224]. The majority of journals and funders now have data sharing policies. National and international data protection laws restrict data sharing by genomic researchers but a number of initiatives have been developed to promote successful data sharing including those hosted by the European Molecular Biology Laboratory’s European Bioinformatics Institute [225], the International Cancer Genome Consortium’s project [226], the Pan-Cancer Analysis of Whole Genomes (PCAWG) [227] and the Human Cell Atlas [228]. The researchers involved in setting up PCAWG have called for an international code of conduct to overcome issues with data protection and provide guidelines for researchers [229].

## Conclusion

The search for discrete genetic, immunological, metabolic, neurological/neurophysiological and behavioural associations with ASD continues [32]. Differential expression of ncRNA genes have shown much promise in various conditions and may be playing a role in the multifactorial aetiology of ASD. At present, no clear conclusions can be drawn from this systematic review for implementation into clinical practice. The key recommendations from our study are to improve research methodologies, reporting and data sharing in this field and to fund and deliver larger studies with more power that will increase the likelihood of being able to answer important questions.

## Supporting information

S1 Fig Gene Ontology analysis heatmap using four most notable differentially expressed miRNA genes in ASD identified by this systematic review. DIANA-miRPath v3.0 online interface DIANA-microT-CDS was used to perform analysis of Gene Ontology Categories (x axis) versus the four key miRNA genes identified in this systematic review (*miR-106b-5p*, *miR-328-3p*, *miR-146a-5p* and *miR-155-5p*) (y axis). P-value and microT threshold were set at < 0.05 and 0.8, respectively and False Discovery Rate (FDR) applied. The heatmap shows the levels of enrichment as determined by Log(p values).
(TIF)

S1 Table Quality assessment using adapted QUADAS-2. (DOCX)

S2 Table Overview of ncRNA gene expression profiles in ASD from all included studies. (XLSX)
