# Supplementary figures and images for: A systematic review of non-coding RNA genes with differential expression profiles associated with autism spectrum disorders
Source: PLoS One. 2023 Jun 15;18(6):e0287131. doi: 10.1371/journal.pone.0287131 (PMC10270643; doi:10.1371/journal.pone.0287131)

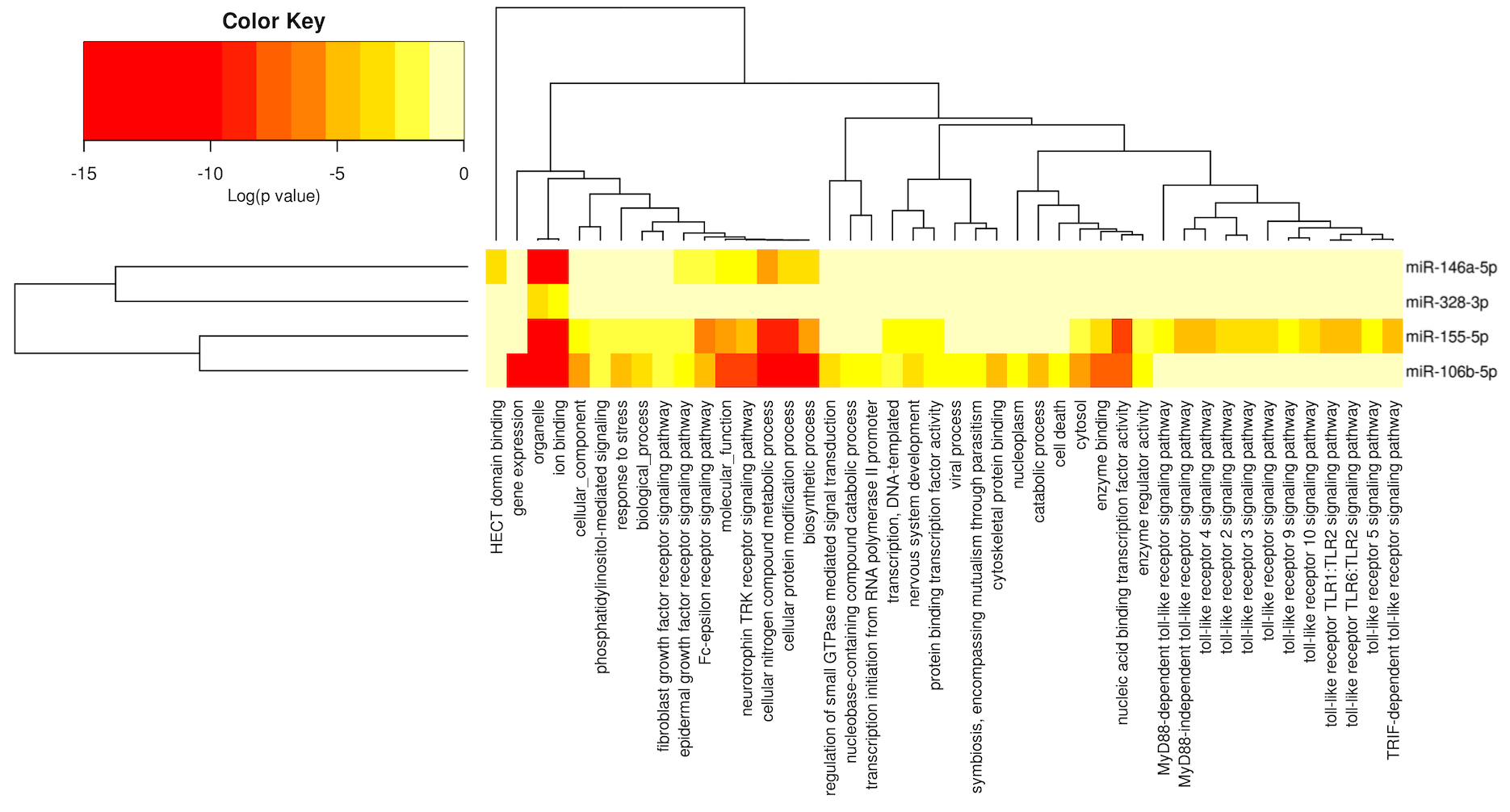

Supplement: S1 Fig — DIANA-miRPath v3.0 online interface DIANA-microT-CDS was used to perform analysis of Gene Ontology Categories (x axis) versus the four key miRNA genes identified in this systematic review (miR-106b-5p, miR-328-3p, miR-146a-5p and miR-155-5p) (y axis). P-value and microT threshold were set at < 0.05 and 0.8, respectively and False Discovery Rate (FDR) applied. The heatmap shows the levels of enrichment as determined by Log(p values). (TIF) [file pone.0287131.s001.tif]
