# Supplementary material for: A systematic review of non-coding RNA genes with differential expression profiles associated with autism spectrum disorders
Source: PLoS One. 2023 Jun 15;18(6):e0287131. doi: 10.1371/journal.pone.0287131 (PMC10270643; doi:10.1371/journal.pone.0287131)
Supplement: S1 Table — (DOCX) [file pone.0287131.s002.docx]

| **Study** | **Participant Selection** | **ASD Assessment** | **ncRNA Measurement** | **Patient Flow** | **Statistical Methodology** |
| --- | --- | --- | --- | --- | --- |
|  | **Risk of Bias (Low / High / Uncertain)** | | | | |
| Abdelrahman et al., 2021 [89] | High | Low | High | Unclear | High |
| Abu-Elneel et al., 2008 [112] | High | High | High | Unclear | High |
| Almehmadi et al., 2020 [79] | High | High | Low | High | High |
| Ander et al., 2015 [85] | High | High | High | Low | High |
| Atwan et al., 2020 [90] | High | High | High | High | High |
| Bleazard, 2017 [113] | High | Low | Low | Unclear | High |
| Cheng et al., 2020 [84] | High | Unclear | High | High | High |
| Cirnigliaro et al., 2017 [91] | Low | Low | High | High | High |
| Cui et al., 2021 [92] | High | High | High | High | Unclear |
| Eftekharian et al., 2019 [93] | High | Low | High | High | High |
| Frye et al., 2021 [114] | High | High | High | High | High |
| Gandal et al., 2018 [87] | High | High | High | High | Unclear |
| Gao et al., 2021 [94] | High | High | High | Unclear | High |
| Hicks, Ignacio et al., 2016 [95] | High | Low | High | Low | High |
| Hicks, Carpenter et al., 2020 [96] | Low | Low | Low | Low | Low |
| Hirsch et al., 2018 [97] | High | Low | High | High | High |
| Huang et al., 2015 [98] | Unclear | Unclear | Low | Unclear | High |
| Jyonouchi et al., 2017 [99] | High | Low | High | Unclear | High |
| Jyonouchi et al., 2019 [100] | High | Low | Unclear | High | High |
| Kichukova, Petrov et al., 2021 [101] | High | Low | High | Unclear | Unclear |
| Kichukova, Popov et al., 2017 [76] | Unclear | Low | High | Unclear | Unclear |
| Moore et al., 2019 [115] | High | High | Low | Unclear | High |
| Mor et al. 2015 [78] | High | High | Low | Low | High |
| Nakata et al., 2019 [102] | High | Low | Unclear | Unclear | High |
| Nguyen, Lepleux et al. 2016 [71] | High | Unclear | High | Unclear | High |
| Nguyen, Fregeac et al., 2018 [82] | High | High | Low | High | High |
| Ozkul et al., 2020 [103] | High | High | Low | High | High |
| Pagan et al., 2017 [72] | High | High | Low | Low | Low |
| Popov, Minchev et al., 2018 [77] | Low | Low | Low | Unclear | High |
| Popov, Minkov et al., 2021 [104] | High | High | Low | High | High |
| Popov & Petrov 2021 [105] | Unclear | Low | High | High | High |
| Ragusa et al., 2020 [81] | High | Low | Unclear | Unclear | High |
| Salloum-Asfar et al., 2021 [73] | High | Low | High | High | High |
| Sarachana et al., 2010 [116] | High | Low | Unclear | High | High |
| Sehovic et al., 2020 [106] | High | High | Low | High | High |
| Sell et al., 2020 [107] | High | High | High | High | Unclear |
| Seno et al., 2011 [117] | High | Low | Low | Unclear | High |
| Shen et al., 2016 [108] | Unclear | High | Unclear | Unclear | Low |
| Stamova et al., 2015 [86] | High | High | Low | High | Low |
| Talebizadeh et al., 2008 [83] | Unclear | Low | Low | Unclear | High |
| Vaccaro et al., 2018 [109] | High | Unclear | Low | Unclear | High |
| Vachev et al., 2013 [110] | High | High | High | Low | High |
| Vasu et al., 2014 [74] | High | Low | Low | High | High |
| Wright et al., 2017 [43] | High | High | Low | Low | High |
| Wu et al., 2016 [80] | High | High | Low | High | Low |
| Yu et al., 2018 [75] | High | High | High | High | High |
| Zamil et al., 2020 [111] | High | High | Low | High | Unclear |
| Zhou et al., 2019 [88] | High | High | Unclear | Unclear | High |

**Table S1. Quality assessment using adapted QUADAS-2.**
